# Network Crosstalk as a Basis for Drug Repurposing

**DOI:** 10.3389/fgene.2022.792090

**Published:** 2022-03-08

**Authors:** Dimitri Guala, Erik L. L. Sonnhammer

**Affiliations:** ^1^ Science for Life Laboratory, Department of Biochemistry and Biophysics, Stockholm University, Solna, Sweden; ^2^ Merck AB, Solna, Sweden

**Keywords:** drug repurposing, drug repositioning, network-based, benchmark, functional association network, network crosstalk, shortest path

## Abstract

The need for systematic drug repurposing has seen a steady increase over the past decade and may be particularly valuable to quickly remedy unexpected pandemics. The abundance of functional interaction data has allowed mapping of substantial parts of the human interactome modeled using functional association networks, favoring network-based drug repurposing. Network crosstalk-based approaches have never been tested for drug repurposing despite their success in the related and more mature field of pathway enrichment analysis. We have, therefore, evaluated the top performing crosstalk-based approaches for drug repurposing. Additionally, the volume of new interaction data as well as more sophisticated network integration approaches compelled us to construct a new benchmark for performance assessment of network-based drug repurposing tools, which we used to compare network crosstalk-based methods with a state-of-the-art technique. We find that network crosstalk-based drug repurposing is able to rival the state-of-the-art method and in some cases outperform it.

## 1 Introduction

Drug repurposing or repositioning has seen a steady increase over the past decade, culminating in a doubling of the number of published articles in PubMed during 2020 (959) compared with 2019 (458). Increased time and cost associated with traditional forms of drug discovery as well as the increase in data for drug repurposing methods are driving the observed rise (Jourdan et al., 2020). The exponential increase seen during the last year can primarily be attributed to the start of the COVID-19 pandemic that launched the scientific community into a desperate search for a quick and affordable solution to the crisis (Singh et al., 2020).

The initial repurposing approaches, during 2020, turned to clinical experience and anecdata-driven predictions, resulting in futile clinical trials investigating the same treatments, such as hydroxychloroquine (Gysi et al., 2021). However, as the panic subsided, a more systematic approach to drug repurposing involving computational drug repurposing methods started to gain more traction. Taking advantage of the increased availability of functional interaction data such as protein–protein interactions (PPIs) and co-regulation in pathways of biological processes, network-based approaches are shown to excel at the drug repurposing task (Guney et al., 2016).

At its core, network-based drug repurposing relies on some form of topological similarity between the set of disease-related proteins and the proteins targeted by a drug in the context of a PPI network. The network in question is the amalgamation of knowledge about the complex biology of the cellular mechanisms involved in the pathology of the studied disease and the mechanism of action of the drugs intended for repurposing. It is, therefore, imperative that the network used for the network-based approaches is comprehensive and biologically sound (Guala et al., 2019).

A previous study of drug repurposing methods (Guney et al., 2016) used a network that consisted of a collection of PPIs, retrieved from a few databases, to benchmark different ways to measure topological similarity and compared the best approach to other state-of-the-art computational drug repurposing methods. The network-based approach relying on the shortest path between the drug-targeted proteins and the closest disease-related protein outperformed other approaches, such as those based on the network distance between targets, network diffusion, chemical similarity, Gene Ontology (Ashburner et al., 2000) term overlap for drug–drug similarity, co-regulation of genes in LINCS (Stathias et al., 2020), and shared side effects.

An adjacent area of bioinformatics that relies on robust and comprehensive functional association networks is functional enrichment analysis, also known as pathway analysis (Maleki et al., 2020). Pathway analysis is often used to uncover the underlying biological mechanisms and processes mediating the effects of drugs, and pathology in diseases. The newest methods, in pathway analysis, study network crosstalk, i.e., the interaction between sets of genes in the network, for example, the crosstalk between a set of disease-related genes and pathways with known biological functions. Given the substantial performance increase of crosstalk-based pathway analysis compared with other approaches (Ogris et al., 2017; Castresana-Aguirre and Sonnhammer, 2020), we propose to use crosstalk-based tools also for drug repurposing. One way to achieve this is to evaluate the similarity between drug-target proteins and disease-related proteins by measuring the crosstalk between the two sets compared with an expected crosstalk in the underlying network between similar sets of nodes in terms of size and connectivity. Another approach would be to use network diffusion (Köhler et al., 2008), also commonly used for network-based pathway analysis (Glaab et al., 2012), to assess drug–disease similarity. However, a network diffusion approach in which longer paths are down-weighted exponentially was already evaluated for drug repurposing and was outperformed by the shortest path approach previously (Guney et al., 2016). Additionally, the same shortest path–based approach performed better than a random walk–based diffusion method in an evaluation of drug repurposing for COVID-19 (Gysi et al., 2021).

The need for reliable computational drug repurposing methods was clear during the outbreak of the COVID-19 pandemic. Since the previous evaluation of drug repurposing methods (Guney et al., 2016), new methods have been developed, more data on drugs and their actions have become available, and the networks of functional associations have become more comprehensive (Guala et al., 2019). Given that new data only partially is used in the form of an updated network in the evaluation of drug repurposing for COVID-19 (Gysi et al., 2021), a complete update of drug repurposing evaluation is highly relevant. A further issue with the Guney et al. (2016) benchmark is that it assumes all unknown drug–disease associations to be negatives, which is unnecessarily strict as some of them are yet to be discovered positives that can be identified by overlap. Taken together, this warrants an update of the benchmark in terms of both data and methodology. In this study, we, therefore, compare the performance of network crosstalk-based drug repurposing with the shortest path–based method considered to be the state of the art on a much larger drug–disease interaction data set in the context of a comprehensive functional association network.

## 2 Materials and Methods

### 2.1 The Guney2016 Benchmark

To evaluate the performance of the studied drug repurposing tools, the data set initially published by Guney et al. was used (Guney et al., 2016). In that data set, disease-associated genes were retrieved from the Online Mendelian Inheritance of Man, OMIM (Hamosh et al., 2005) database and the GWAS catalog at PheGenI (Ramos et al., 2013), keeping 79 diseases with at least 20 genes in the underlying human PPI network from a previous study (Menche et al., 2015). The network comprised a collection of PPIs from a few experimental databases consisting of 13,329 proteins and 141,150 interactions. Drugs and drug targets were downloaded from DrugBank (Wishart et al., 2018) and mapped to diseases using medical subject headings. Drugs without targets in the underlying network as well as drugs with the same therapeutic indication and targets as already included drugs were removed, yielding 238 drugs. This produced a benchmark of 402 positive and 18,162 negative drug–disease pairs.

### 2.2 The FunCoup Benchmark

#### 2.2.1 The Functional Association Network

One of the most comprehensive functional association networks, FunCoup (Alexeyenko et al., 2011; Persson et al., 2021), was used as the substrate for the network-based drug repurposing algorithms in this benchmark. FunCoup is a framework for integrating large-scale experimental data on gene–gene and protein–protein interactions. It uses naïve Bayesian integration to combine both direct and indirect interactions stemming from 11 different types of data, pertaining to co-expression, co-localization, co-evolution, co-regulation, domain–domain interaction, genetic interaction, and PPI (Guala et al., 2019). Each type of evidence is uniquely scored, shared via orthology across the included species, and trained using gold standards, such as metabolic and signaling pathways from Kyoto Encyclopedia of Genes and Genomes (KEGG) (Kanehisa, 2000), confirmed pairwise and complex PPIs from iRefIndex (Razick et al., 2008), CORUM (Giurgiu et al., 2019), and ComplexPortal (Meldal et al., 2019) as well as operon information from OperonDB (Pertea et al., 2009). The latest update of FunCoup, release 5, which was used in the study, encompasses genome-wide networks for 21 different species from all domains of life and the SARS-CoV2 virus–human interactome (Persson et al., 2021); however, only its *Homo sapiens* network was used in this study. The default interaction confidence threshold of 0.8, applied to enrich the resulting network for high quality interactions, generated a *H. sapiens* network containing 12,848 proteins and 612,284 interactions.

#### 2.2.2 Disease and Drug Target Data

The Comparative Toxicogenomic Database (CTD) (Davis et al., 2021) was used as the source of manually curated information about drugs and diseases including drug–target interactions as well as disease-related genes, or disease genes in short. Drug target information in CTD is manually curated from published literature as well as public databases, such as DrugBank. The information on diseases and related genes is also manually curated from published literature or extracted from OMIM. For the purpose of this study, the initial set of CTD drugs was filtered for drugs with at least one drug target in the FunCoup network, resulting in 1370 drugs. A similar filtering was performed for the CTD diseases. However only diseases with at least 20 disease-related genes in the FunCoup network were kept as in similar evaluations (Guney et al., 2016), yielding 212 well-characterized diseases. The combination of diseases and drugs comprised 8518 drug–disease associations, which were designated as positive in the continued analysis. The remaining 281,922 drug–disease combinations were designated as negative.

For a negative drug–disease combination with an overlap between drug targets and disease genes, the studied drug–disease pair may be positive but yet unverified. To minimize the risk of misclassifying a positive drug–disease combination as a false positive, all combinations with gene overlap were removed from the negatives set. The final benchmark contained 8518 positive and 159,144 negative drug–disease pairs.

### 2.3 The Time-Stamped Benchmark

To validate newly predicted drug–disease combinations, we used new indications that have come about after the Guney2016 benchmark and used these together with the original network to construct a time-stamped benchmark. There were 40 diseases that could be matched by name in both the Guney2016 benchmark and the FCbench. Drugs that have been repurposed since the Guney2016 benchmark, i.e., drugs that received a new indication for at least one of the 40 diseases, caused a change in the label of the corresponding drug–disease combinations, switching from negative status in the Guney2016 benchmark to positive status in the time-stamped benchmark. This produced 362 new drug–disease combinations. After removing 473 negatives due to overlap between drug targets and disease genes, the time-stamped benchmark comprised 764 positive and 17,327 negative drug–disease pairs.

### 2.4 Network-Based Drug Repurposing Methods

**FIGURE 1:**
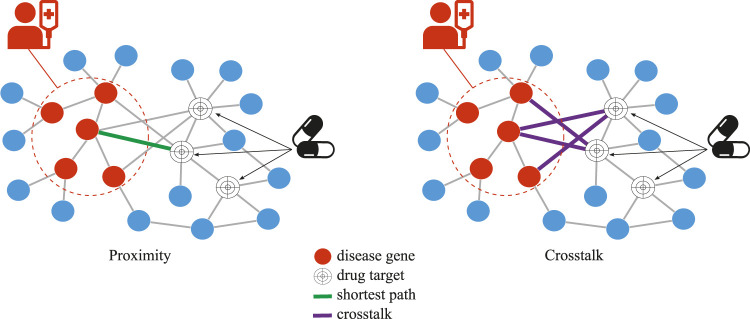
Network-based drug repurposing, proximity vs. crosstalk, for a given disease and drug. In crosstalk, the number of network links between all drug targets and disease genes is used while, in proximity, the length of the single shortest path between any drug target and any closest disease gene is used.

Network-based drug repurposing methods in this study used either shortest path or network crosstalk to assess the similarity between the set of drug targets and the set of disease genes (Figure 1). There are different ways of measuring the shortest path between two sets of nodes in a network. In a previous study (Guney et al., 2016), the shortest path between drug targets and the closest disease gene is shown to outperform the other distance measures. We, therefore, used that method in our study and refer to it as “proximity.” Because crosstalk-based distance measures have never before been used for drug repurposing, we used the three most prominent crosstalk-based approaches here.

#### 2.4.1 Proximity

To assess the distance, d , between a drug, T , and a disease, S , the shortest path between all drug targets and all disease-related proteins was calculated. The distance, d(T, S) , was the average shortest path between every drug target and its closest disease gene, where ||T|| is the number of drug targets.

$$d\left(T,\;S\right)=\frac{1}{\|T\|}\sum_{t\in T}min_{s\in S}d\left(t,\;s\right)$$

To normalize the distance with respect to the topological properties of the network, a degree-aware node permutation approach was adopted. The nodes in the network were divided into bins according to node degree with 100 nodes in each bin. A random sample of drug targets and disease genes was performed, matching the sampled nodes in both the number and degree distribution by sampling nodes from the same bin as the observed targets/genes. The d(T,S) was calculated for the sampled sets and the procedure was repeated 1000 times. The mean, $\mu_{R}$ , and standard deviation, $\sigma_{R}$ , of the distribution of randomly sampled distances was used to normalize the observed distance and to assess its significance. (Guney et al., 2016).

$$z_{prox}=\frac{d-\mu_{R}}{\sigma_{R}}$$

Proximity was sped up and re-implemented in R for the purpose of this study from the original Python version (https://github.com/emreg00/proximity) published by Guney et al. (2016).

#### 2.4.2 NEAT

For this drug repurposing method, similarity between two sets of nodes in a network is estimated by measuring the crosstalk, i.e., the number of links, between the two sets. A larger crosstalk suggests a greater similarity between the two sets. To normalize the observed crosstalk, x , the mean, $\mu_{H}$ , and the standard deviation, $\sigma_{H}$ , of the expected crosstalk between sets of nodes with the same size as the drug targets and disease genes can be calculated assuming a hypergeometric distribution of links in the underlying functional association network (Signorelli et al., 2016). Below the number of drug targets, K , the number of disease genes, n , and the total number of genes in the network, N , can be used to determine, the mean, $\mu_{H}$ , and the standard deviation, $\sigma_{H}$ , $\mu_{H}=n\frac{K}{N}$ and $\sigma_{H}=\sqrt{\mu_{H}\frac{\left(N-K\right)}{N}\frac{N-n}{N-1}}$ .

Normalization of crosstalk is carried out as follows:

$$z_{NEAT}=\frac{x-\mu_{H}}{\sigma_{H}}$$

#### 2.4.3 BinoX

The BinoX approach to drug repurposing also uses crosstalk as a measure of similarity, but assumes a binomial distribution, i.e., $Bin\left(n_{x},\;p_{x}\right)$ , of crosstalk between two sets of nodes in the underlying network (Ogris et al., 2017), where $n_{x}$ is the maximum possible crosstalk. To estimate the parameter $p_{x}$ of the underlying distribution, to serve as a comparator for the observed crosstalk, x , the network is randomized M times using topology-preserving link shuffling (McCormack et al., 2013).

$$p_{x}=\frac{\frac{1}{M}\sum_{i}^{M}x_{i}}{n_{x}}$$

The mean, $\mu_{b}=n_{x}p_{x}$ , and the standard deviation, $\sigma_{b}=n_{x}p_{x}\left(1-p_{x}\right)$ can be calculated for the expected crosstalk, using $n_{x}$ and $p_{x},$ and the observed crosstalk can be normalized producing a *z*-score as for NEAT.

For calculating $\mu_{b}$ and $\sigma_{b}$ , the stand-alone version of the pathway enrichment tool BinoX (https://bitbucket.org/sonnhammergroup/binox/) was used (Ogris et al., 2017).

#### 2.4.4 ANUBIX

The ANUBIX approach assumes that network crosstalk follows a beta-binomial distribution, where the probability of crosstalk is binomially distributed with the parameter $\;p_{x}$ following a Beta distribution, i.e., Beta(α,β) . To compare the observed crosstalk to the expected crosstalk, a degree-aware randomization procedure is applied. In this procedure, for each drug–disease pair, a set of nodes with the same size and degree distribution as the set of drug targets is sampled from the network, and its crosstalk with the set of disease genes is calculated. The procedure is repeated 100 times, and maximum likelihood estimation is used to estimate the parameters of the Beta distribution. The mean and standard deviation of the sampled crosstalk are calculated as for *BinoX* and a similar normalization procedure is applied to obtain the normalized crosstalk score, $z_{anubix}$ . The estimation of parameters for the Beta distribution as well as the calculation of mean, standard deviation, and *p*-values was conducted using a re-implementation of the pathway enrichment tool ANUBIX (Castresana-Aguirre and Sonnhammer, 2020).

### 2.5 Drug–Drug Similarity–Based Repurposing

For diseases that already have approved treatment options, a common repurposing technique is to study the similarity of new drugs to the already approved ones. We, therefore, applied the above-mentioned repurposing methodologies to assess drug–drug similarity of new drugs to the drugs already approved for a disease.

For proximity, the drug–drug similarity, s(T, S) , between drug T and disease S is

$$s_{prox}\left(T,S\right)=e^{-min\left(z_{T,S}\right)}$$

For the crosstalk-based methods, the drug–drug similarity is

$$s_{crosstalk\;}\left(T,S\right)=max\left(z_{T,S}\right)$$

The z vector in both equations above refers to the method-specific normalized *z*-scores calculated between drugs indicated for a disease and each of the other candidate drugs, where $T_{s}$ denotes drugs approved for disease S with $t_{s}$ being all the drug targets for the drug $T_{s}$ .

$$z_{T,S}=\forall_{T_{s}\;approved\;S}z\left(t\in T,t_{s}\in T_{s}\right)$$

### 2.6 Performance Evaluation

Prior to running the drug repurposing methods on the drug–disease combinations in the three data sets, we examined the set of negative drug–disease combinations for potential positives misclassified as negatives. The potential positives were deemed to be those drug–disease combinations for which there was gene overlap between drug targets and disease genes. Potential positives were filtered from the analysis.

Running each drug repurposing approach on every drug–disease combination produced *z*-scores and corrected *p*-values, which were used in the evaluation. To interpret the *z*-score output from a drug repurposing method as a classification of drug–disease combinations into positives or negatives, a *z*-score threshold was required. In the Guney2016 benchmark, the threshold was set as the *z*-score at the intersection of the sensitivity and specificity curves when visualizing the change in sensitivity and specificity as functions of the *z*-score in the same graph. Sensitivity was defined as the proportion of correctly assigned instances of the positive class, also known as recall or true positives among all positives. Specificity was defined as the proportion of correctly assigned instances of the negative class, also known as true negatives among all negatives.

Performance metrics used in the Guney2016 benchmark and in this study are considered to be among the most commonly used performance metrics for evaluation of drug repurposing approaches (Schuler et al., 2020) and include receiver operating characteristic (ROC) curve and area under the ROC curve, AUROC . The ROC curve is a convenient visualization of the change in sensitivity also known as recall or true positive rate, TPR , and the false positive rate, FPR , at various thresholds, T . The AUROC provides a summary metric for the performance of a classifier visualized in the ROC curve and can be interpreted as the probability of ranking a randomly chosen positive sample higher than a randomly chosen negative one (Bradley, 1997).

$$AUROC=\overset{\infty }{\underset{-\infty }{\int }}TPR\left(T\right)FPR\prime \left(T\right)dT$$

AUROC was calculated both for the full data set of all drug–disease combinations as well as on 100 randomly sampled sets containing all positives and equally many negatives. The sampling procedure was applied to address the inherent class imbalance of the data set. The Wilcoxon signed-rank test was used to compare the performance of different drug repurposing methods.

Additionally, we also assessed the precision recall (PR) curve as well as the area under it (AUPR) (Keilwagen et al., 2014) because both are also commonly used to assess performance of classifiers. The PR curve visualizes the change in precision and recall at different thresholds. Because the number of true positive drug–disease combinations is much lower than the number of false positive ones, the AUPR punishes the top-ranked FPs more than AUROC (Davis and Goadrich, 2006). However, the whole premise of drug repurposing is that our knowledge of valid drug–disease combinations is incomplete, meaning that many of the drug–disease combinations that we currently consider as false positive are merely untested true positives. This notion is supported by both the time-stamped benchmark and other work (Rivero-García et al., 2021) and is causing the AUPR to be an unreasonably conservative and, thus, less suitable performance measure as compared with AUROC.

The abovementioned metrics rely on the knowledge of both true positives and true negatives. However, most of the drug–disease combinations designated as negatives are, in fact, unknown. Although we did try to address this by filtering combinations with overlap in drug targets and disease genes, there may still be many potential positives left. We, therefore, also assessed performance of drug repurposing using the less rigorous metric of the recall at the *z*-score cutoff (Brown and Patel, 2018).

### 2.7 Multiple Hypothesis Testing

All *p*-values produced in the study were corrected for multiple hypothesis testing using the Benjamini–Hochberg procedure (Benjamini and Hochberg, 1995).

## 3 Results

### 3.1 Benchmark Characteristics

The benchmark presented here comprises a total of 290,437 drug–disease combinations, where 8,397 came from approved indications, 180 from clinical trials, 242 were contraindicated, and 121 were registered as off-label. The remaining 281,500 came from linking drugs to diseases with which they are not associated, denoted as “unknown.” Drug–disease pairs with approved indication or used off-label were considered as positives. Contraindicated combinations and drug–disease pairs in clinical trials were removed. The remaining drug–disease combinations, i.e., the unknown, were denoted as negatives. Sensitivity analysis when adding back contraindicated drug–disease pairs as well as pairs studied in clinical trials to the positive class was conducted and yielded the same results (Supplementary Figure S1).

**FIGURE 2:**
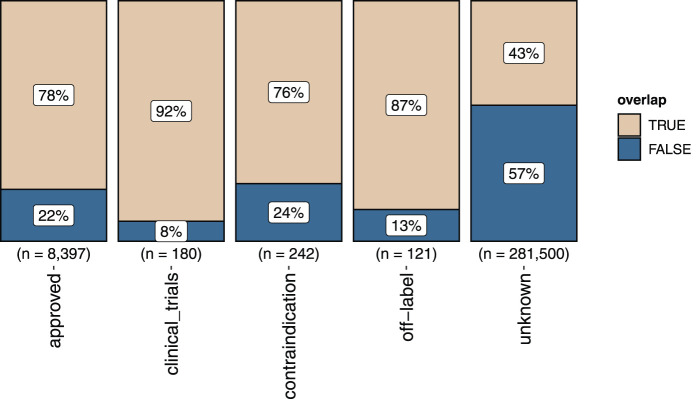
The proportion of gene overlap (marked “TRUE”) between drug targets and disease genes in the different sets of positive and negative drug–disease combinations. The proportion of not overlapping genes is marked “FALSE”.

For a negative drug–disease combination with an overlap between drug targets and disease genes the studied drug–disease pair may be positive but yet unverified. This is supported by the fact that the proportion of drug–disease combinations with overlapping genes was markedly enriched for approved or off-label annotations compared with those annotated as “unknown” (Figure 2). Removing drug–disease combinations with gene overlap from the negative set resulted in the FCbench containing 8,518 positives and 159,144 negatives. This represents a 21-fold increase of positives and a nine-fold increase of negatives compared with the Guney2016 benchmark.

A subset of 40 diseases that appeared in both the Guney and the current benchmarks had received at least one new drug as a treatment. We, therefore, updated the classification of these new drug–disease combinations in the Guney2016 benchmark from negative to positive to produce a time-stamped benchmark. The time-stamped benchmark comprises 764 positives and 17,327 negatives.

### 3.2 Evaluation of Methods to Score Drug–Disease Association–Based Drug Repurposing

The relationship between drug targets and disease genes in the context of a functional association network was assessed using four different drug repurposing approaches: one based on the shortest paths, proximity, and three based on crosstalk, NEAT, BinoX, and ANUBIX. Since the studied relationship was between drug targets and disease genes, we refer to the outcome as a drug–disease association. Each of the evaluated drug repurposing approaches produced an association score between a drug and a disease with higher score, i.e., shorter distance for proximity and higher crosstalk for the other methods, suggesting a higher probability of the studied drug being used for the specific disease. The scoring was evaluated on three data sets with drug–disease combinations, using ROC curves, AUROC, and recall (see *Materials and Methods*). PR curves, AUPR, and some other metrics, e.g., Matthews correlation coefficient (mcc) and F1-score, were also evaluated for completeness, but due to their unsuitability as performance metrics for this task were only included in the Supplementary Material.

#### 3.2.1 Performance on the Guney2016 Benchmark

**FIGURE 3:**
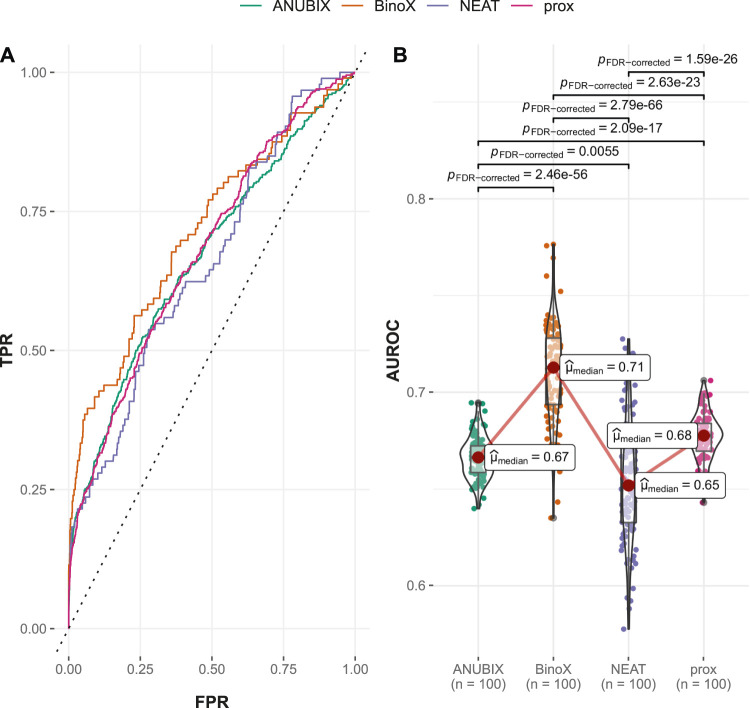
Drug–disease association–based performance on the Guney2016 benchmark. Performance of the different drug repurposing tools: ANUBIX (green), BinoX (orange), NEAT (purple), and proximity (prox, pink) on **(A)** ROC curves, where the dotted line represents random prediction, and **(B)** AUROC using sampled sets from the benchmark containing equal numbers of positive and negative drug–disease combinations. The pairwise Wilcoxon rank sum test was used to assess the significance of difference on AUROC. FDR-corrected *p*-values were obtained using the Benjamini–Hochberg procedure.

Visual inspection of the ROC curves for the full Guney2016 data set suggests superior performance of the BinoX approach compared with all the other approaches (Figure 3A). The AUROC achieved by BinoX (median = 0.714) stands out from the rest that tightly follow each other: proximity (median = 0.677), ANUBIX (median = 0.667), and NEAT (median = 0.657) (Table 1). The AUROC under the equal label sampling from the benchmark confirms the order and indicates that the observed differences in performance were statistically significant (*p* < .01).

**TABLE 1:** Performance metrics for all three benchmarks. Performance on the three benchmarks, Guney 2016, time-stamped (time), and FCbench, was assessed for different drug repurposing tools: ANUBIX, BinoX, NEAT, and proximity (prox). The evaluated metrics include AUROC, recall, and recall on the pairs with changed label in the time-stamped benchmark (new positives).

	AUROC			recall			
	Guney2016	Time	FCbench	Guney2016	Time	Time (new positives)	FCbench
ANUBIX	0.667	0.645	0.849	0.433	0.386	0.334	0.782
BinoX	0.714	0.541	0.728	0.635	0.126	0.094	0.672
NEAT	0.657	0.535	0.759	0.602	0.120	0.099	0.694
prox	0.677	0.633	0.868	0.493	0.425	0.351	0.793

#### 3.2.2 Performances on the Time-Stamped Validation

**FIGURE 4:**
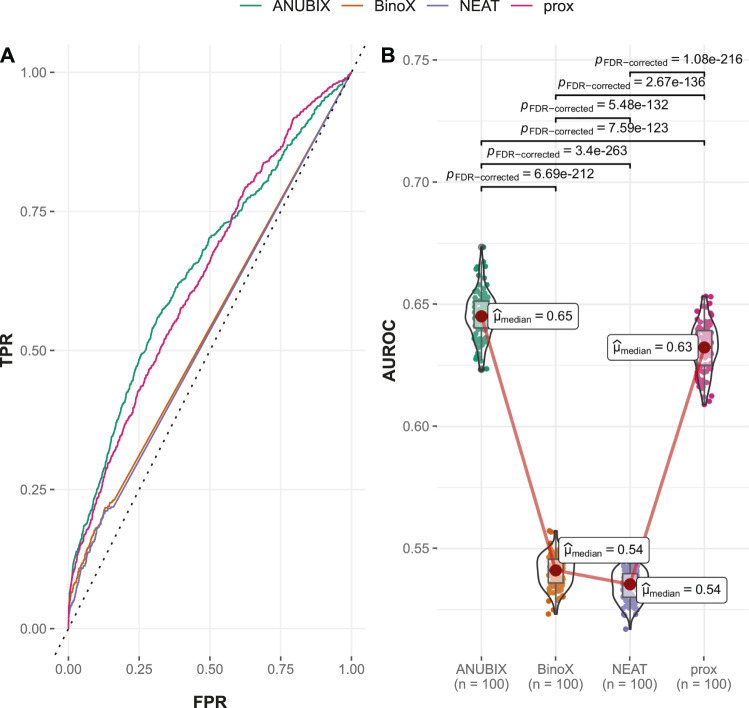
Drug–disease association–based performance on the time-stamped benchmark. Performance of the different drug repurposing tools: ANUBIX (green), BinoX (orange), NEAT (purple), and proximity (prox, pink) on **(A)** ROC curves, where the dotted line represents random prediction, and **(B)** AUROC using sampled sets from the benchmark containing equal numbers of positive and negative drug–disease combinations. The pairwise Wilcoxon rank sum test was used to assess the significance of difference on AUROC. FDR-corrected *p*-values were obtained using the Benjamini–Hochberg procedure.

In the time-stamped validation of the Guney2016 benchmark, 362 drug–disease associations were changed from negatives to positives, resulting in almost a doubling of the number of positive combinations from 402 to 764. This resulted in a variable degree of loss of performance for all the methods (Table 1). The largest drop in AUROC performance was noted for BinoX by 17% (AUROC = 0.541) followed by NEAT 12% (AUROC = 0.535). As a result, BinoX no longer outperformed the other methods, and instead, the top methods became ANUBIX (AUROC = 0.645) and proximity (AUROC = 0.633), which were considerably more accurate. The difference in performance between the methods was once again statistically significant (Figure 4).

The explanation for the large drop in performance for BinoX and NEAT is the fact that both methods correctly classified only roughly 10% of the new positives as positives while ANUBIX and proximity correctly classified 33% and 35% of the new positives, respectively (Table 1).

#### 3.2.3 Performances on the FCbench

**FIGURE 5:**
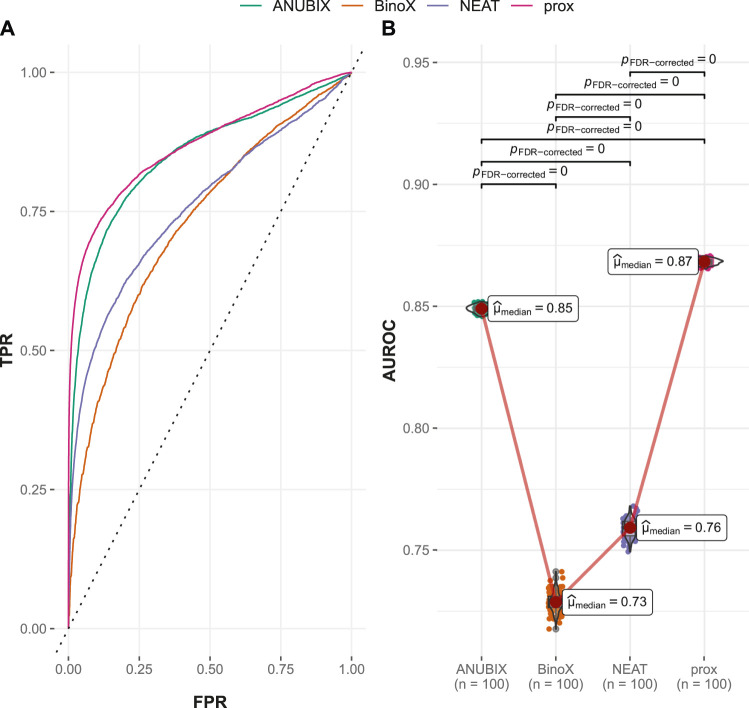
Drug–disease association–based performance on the FCbench. Performance of the different drug repurposing tools: ANUBIX (green), BinoX (orange), NEAT (purple), and proximity (prox, pink) on **(A)** ROC curves, where the dotted line represents random prediction, and **(B)** AUROC using sampled sets from the benchmark containing equal numbers of positive and negative drug–disease combinations. The pairwise Wilcoxon rank sum test was used to assess the significance of difference on AUROC. FDR-corrected *p*-values were obtained using the Benjamini–Hochberg procedure.

The FCbench contains an order of magnitude more positives than the Guney2016 one (8,397 versus 402) and the time stamped one, 764 vs. 402. It is also based on a denser and more complete underlying network, which boosted the performance of all methods (Figure 5). The difference in performance proved once again to be statistically significant with proximity outperforming ANUBIX with an AUROC of 0.868 vs. 0.849, followed by NEAT at 0.759, and finally BinoX at 0.728 (Table 1). A much higher recall was also observed, primarily for proximity and ANUBIX at 0.793 and 0.782, respectively, almost doubling the recall from the Guney2016 and time-stamped benchmarks. NEAT and BinoX had a recall of 0.694 and 0.672, respectively.

Performance of the tested drug repurposing methods differed for the used benchmarks with each benchmark having a different winner. However, since proximity was always ranked as the top or second best method, it could be considered an overall winner with ANUBIX performing equally well if the old benchmark is ignored. Both proximity and ANUBIX performed considerably better than NEAT and BinoX on the benchmarks based on new data.

### 3.3 Application of Predicted Drug–Disease Associations for Drug Repurposing

In the FCbench, the two top-performing methods, ANUBIX and proximity, produced similar AUROC and recall values with a difference of about 2% in AUROC in favor of ANUBIX and 1% in recall in favor of proximity. Although this difference is statistically significant (*p* < .01) for AUROC, it is hardly meaningful in terms of overall performance.

Do these two approaches recover the same or similar sets of drug–disease combinations? The intersection of correctly classified drug–disease combinations for ANUBIX and proximity contained 6,261 elements while each method uniquely correctly classified 404 and 497 drug–disease combinations, respectively; i.e., they overlapped by ∼94%. Despite the relatively high coverage demonstrated by both methods and using the same prior knowledge in the form of the FunCoup network, there were still unique predictions provided by both methods.

To suggest potential drug repurposing opportunities, we investigated the drug–disease combinations assigned as negatives, including combinations previously removed due to overlap between drug targets and disease genes. We chose to focus on predictions from the two top performing methods, ANUBIX and proximity. To increase the chances of identifying potential positives, we set a method-specific *z*-score cutoff, $z_{cutoff}=\mu_{z}+2\sigma_{z}\;$ , only considering combinations with *z*-scores above the cutoff. This produced 5,654 combinations agreed upon by both methods (Supplementary Table S2) as well as 5,967 and 6,302 combinations assigned uniquely by ANUBIX (Supplementary Table S3) and proximity (Supplementary Table S4), respectively. It should be noted that the *z*-score cutoff was higher for ANUBIX (*z* = 8.25) vs. proximity (*z* = 5.89), which can explain the slightly lower number of uniquely retrieved combinations. Among the identified combinations, 97% contained an overlap between drug targets and disease genes.

After sorting the retrieved drug–disease combinations by the highest *z*-scores from proximity and ANUBIX, we looked at the top 10 suggestions. The top suggestion for repurposing was palbociclib (DB09073) for hepatocellular carcinoma (HCC). Palbociclib is a selective inhibitor of cyclin dependent kinases CD4/CD6, used to treat HER2-negative and HR-positive advanced or metastatic breast cancer (Finn et al., 2009). However, it also showed promising results as a novel therapeutic strategy in preclinical models of HCC (Bollard et al., 2017). Only looking at the results from proximity, this combination would have been in 10th place, but adding the prediction from ANUBIX, we were able to identify this combination as the top candidate for repurposing.

The second drug–disease combination on the candidates list was azathioprine (DB00933) and HCC. Azathioprine is an immunosuppressant used to prevent renal transplant rejection as well as in treatment of autoimmune disorders, such as Crohn’s disease and rheumatoid arthritis. Although a conclusive link between azathioprine and HCC has never been established, multiple case reports suggest a possible association (Heron et al., 2016). This finding exemplifies a case when the uncovered drug–disease combination is of a potential side effect nature rather than a treatment effect, which is usually desired. At the same time, this supports previously observed results that network-based drug repurposing can identify potential side effects and negative drug interactions (Badkas et al., 2021).

Third on the list is testosterone (DB00624) and HCC. Testosterone is indicated for treatment of hypogonadism and hypogonadotropic hypogonadism. However, animal models suggest the implication of testosterone in the etiology of HCC, which was later supported by the results from a study in humans where elevated levels of testosterone were associated with increased risk of HCC (Yu and Chen, 1993). This type of association could be considered as disease-biomarker rather than disease treatment or drug–disease side effect. It is not the primary goal of drug repurposing but could potentially open other avenues for the use of network-based approaches.

In the eighth place is the combination of one of the few gold-based drugs, Sodium aurothiomalate (DB09276) and the human influenza. The human influenza is 50%–65% suppressed by the current vaccination program, so there may not be a real need for repurposing any drugs for it. However, there is evidence showing the potential of gold-based treatments against other viruses. One such example is auranofin that inhibits replication of the SARS-COV-2 virus (Rothan et al., 2020), suggesting that gold-based compounds may also be useful against influenza virus.

Looking at the combinations uniquely predicted for repurposing by ANUBIX, we found enalaprilat (DB09477) for left ventricular dysfunction (LVD) as one of the top suggestions with a *z*-score of 66.3 (Supplementary Table S3). Enalaprilat is indicated for treatment of hypertension. However, there is clinical evidence of enalaprilat causing marked reduction of development of atrial fibrillation in patients with LVD (Vermes et al., 2003). In sixth place, the combination of tetrabenazine (DB04844) and Parkinsonian disorders received a *z*-score of 49.9. Tetrabenazine is approved for treatment of Huntington’s disease but is widely used for various movement disorders for which, among other effects, it improves levodopa-induced peak-dose dyskinesias in patients with Parkinson’s disease (Brusa et al., 2013).

### 3.4 Evaluation of Methods to Score Drug–Drug Similarity–Based Repurposing

A common approach in drug repurposing for diseases with already approved drugs is to identify other drugs that are similar to the already approved ones. To this end, we here calculate a score for a drug–disease combination based on the similarity between a drug candidate and a drug already approved for that disease. This similarity is based on either the shortest path or crosstalk between the drug targets of a candidate drug and the drug targets of a drug approved for the disease, resulting in a *z*-score for the pair of drugs. The *z*-scores are then combined across all approved drugs to produce a drug–disease similarity score for the drug candidate (see Methods).

#### 3.4.1 Performances on the Guney2016 Benchmark

**FIGURE 6:**
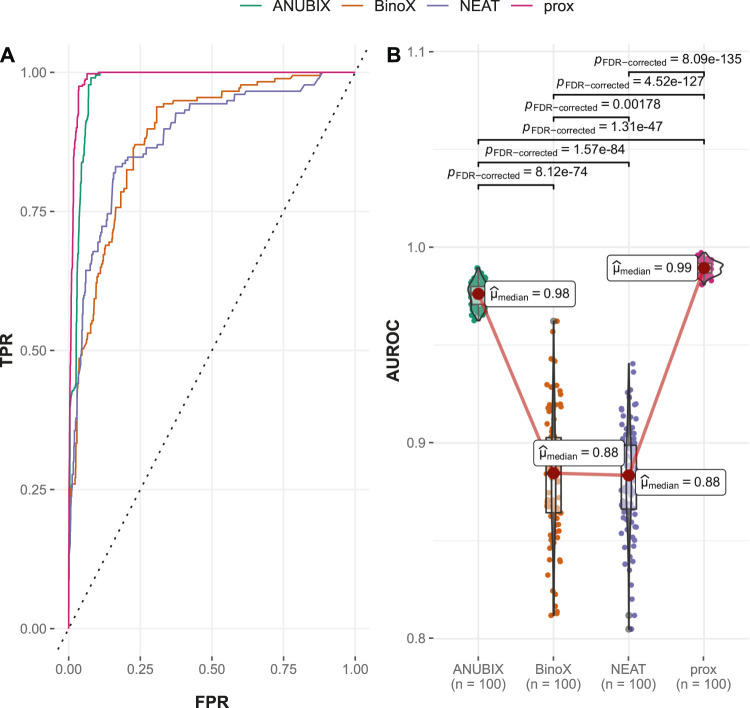
Drug–drug similarity–based performance on the Guney2016 benchmark. Performance of the different drug repurposing tools: ANUBIX (green), BinoX (orange), NEAT (purple), and proximity (prox, pink) on **(A)** ROC curves, where the dotted line represents random prediction, and **(B)** AUROC using sampled sets from the benchmark containing equal numbers of positive and negative drug–disease combinations. The pairwise Wilcoxon rank sum test was used to assess the significance of difference on AUROC. FDR-corrected *p*-values were obtained using the Benjamini–Hochberg procedure.

The ROC and AUROC performance was markedly increased compared with drug–disease association with over 30% improvements for proximity and ANUBIX*,* followed by 22% improvement for NEAT, and 17% for BinoX (Table 1 and Table 2). The results show significantly (*p* < .01) different performance for all pairwise comparisons except between NEAT and BinoX (Figure 6). At an AUROC of 0.989, proximity showed 1.4% higher performance than ANUBIX (AUROC = 0.975), followed by BinoX and NEAT at an AUROC of 0.885 and 0.883, respectively. The recall increased by about twofold compared with the drug–disease evaluation for proximity and ANUBIX*,* resulting in 0.925 and 0.960, respectively, meaning that almost a complete recovery of all positives was achieved. An increased recall was observed for NEAT and BinoX as well.

**TABLE 2:** Drug–drug similarity–based performance metrics for all three benchmarks. Performance on the three benchmarks, Guney 2016, time-stamped (time), and FCbench, was assessed for different drug repurposing tools: ANUBIX, BinoX, NEAT, and proximity (prox). The evaluated metrics included AUROC, recall, and recall on the pairs in the time-stamped benchmark (new positives).

	AUROC			recall			
	Guney2016	Time	FCbench	Guney2016	Time	Time (new positives)	FCbench
ANUBIX	0.975	0.829	0.883	0.925	0.719	0.391	0.823
BinoX	0.885	0.813	0.823	0.791	0.766	0.046	0.740
NEAT	0.883	0.813	0.803	0.831	0.748	0.046	0.732
prox	0.989	0.820	0.973	0.960	0.730	0.414	0.910

#### 3.4.2 Performances on the Time-Stamped Validation

**FIGURE 7:**
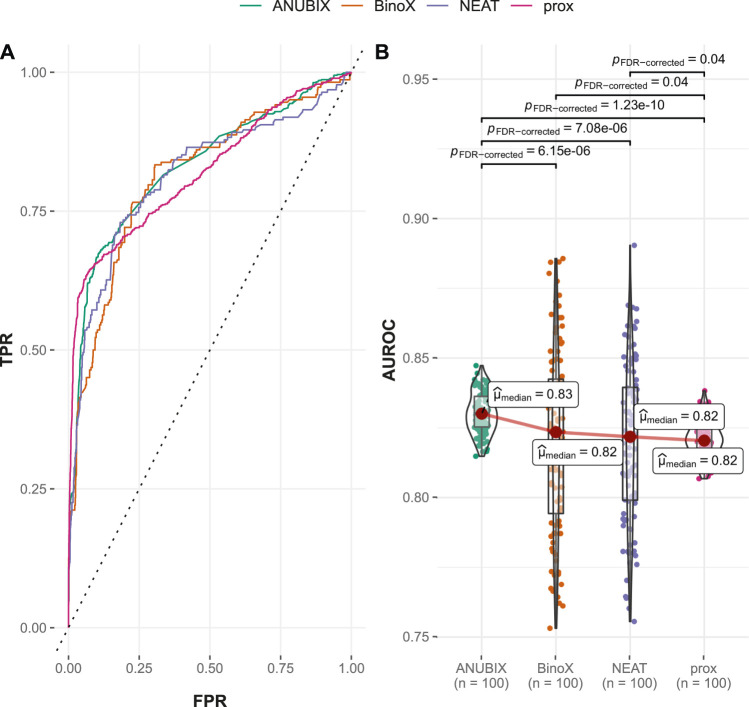
Drug–drug similarity–based performance on the time-stamped benchmark. Performance of the different drug repurposing tools: ANUBIX (green), BinoX (orange), NEAT (purple), and proximity (prox, pink) on **(A)** ROC curves, where the dotted line represents random prediction, and **(B)** AUROC using sampled sets from the benchmark containing equal numbers of positive and negative drug–disease combinations. The pairwise Wilcoxon rank sum test was used to assess the significance of difference on AUROC. FDR-corrected *p*-values were obtained using the Benjamini–Hochberg procedure.

Also for the time-stamped analysis, performance of drug–drug similarity was higher for all studied approaches than when drug–disease association was used. However, in comparison with the performance of drug–drug similarity on the Guney2016 data set, the time-stamped performance was lower, in particular for the previously best-performing tools, proximity and ANUBIX (Table 2). With a 1% margin, ANUBIX significantly outperformed proximity in this analysis with an AUROC of 0.829 vs. 0.820. The difference in AUROC was not statistically significant between proximity, NEAT*,* and BinoX (Figure 7). The recall for the time-stamped data set was markedly lower than in the Guney2016 benchmark for all methods except BinoX, which was only slightly lower. BinoX achieved the highest recall of 0.766 followed by NEAT at 0.748, proximity at 0.730, and ANUBIX at 0.719. Compared with drug–disease association, the recall on the new positives was only marginally higher for proximity and ANUBIX, landing on 0.414 and 0.391, respectively. The already low recall of new positives seen for BinoX and NEAT in the drug–disease association analysis shrank further to 0.046 for drug–drug similarity (Table 2).

#### 3.4.3 Performances on the FCbench

**FIGURE 8:**
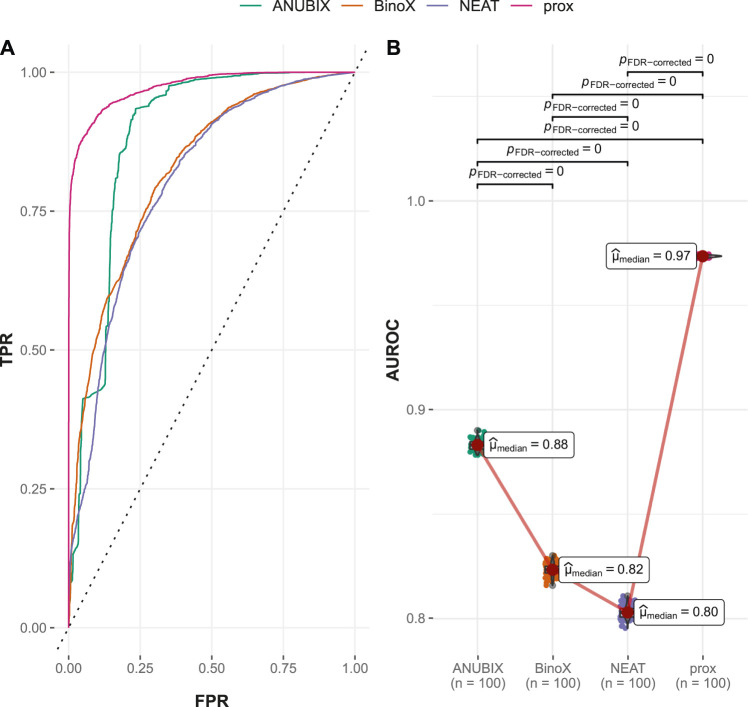
Drug–drug similarity–based performance on the FCbench. Performance of the different drug repurposing tools: ANUBIX (green), BinoX (orange), NEAT (purple), and proximity (prox, pink) on **(A)** ROC curves, where the dotted line represents random prediction, and **(B)** AUROC using sampled sets from the benchmark containing equal numbers of positive and negative drug–disease combinations. The pairwise Wilcoxon rank sum test was used to assess the significance of difference on AUROC. FDR-corrected *p*-values were obtained using the Benjamini–Hochberg procedure.

The drug–drug similarity–based performance on the FCbench also increased compared with the corresponding drug–disease association–based one for all studied approaches. Proximity reached the highest AUROC of 0.973, followed by ANUBIX at 0.883, BinoX at 0.823, and *NEAT* at 0.803 (Figure 8). The same ranking was reflected in the recall values, where proximity, ANUBIX, BinoX, and NEAT reached 0.910, 0.823, 0.740, and 0.732, respectively (Table 2). All differences in AUROC were statistically significant (*p* < .01) (Figure 8).

## 4 Discussion

The last couple of years shows that the possibilities for drug repurposing were far from satisfactory and called for better methods, in particular computational network-based approaches that can combine all existing knowledge of drugs, targets, disease genes, and networks. This study introduces a new approach to network-based drug repurposing based on network crosstalk, rivalling available state-of-the-art methods, together with an updated and comprehensive *in silico* performance assessment platform.

The Guney2016 benchmark for drug repurposing tools introduced by Guney et al. (2016) was applied naïvely but also utilized as the basis for a time-stamped update. Although the BinoX tool outperformed the other approaches on the Guney2016 benchmark, the same achievement was not reproduced in the time-stamped approach nor in the new, more comprehensive FCbench. Except for the ranking on the Guney2016 benchmark, it was clear that the top crosstalk-based approach was *ANUBIX*. This can be attributed to the fact that the both BinoX and NEAT approaches are more prone to false positives compared with ANUBIX due to the beta-binomial distribution being a more accurate model of random crosstalk in the underlying network and that ANUBIX accounts for nonrandom intrapathway interactions (Castresana-Aguirre and Sonnhammer, 2020). An additional insight from the three benchmarks was that all methods performed much better on the FCbench, which can be attributed to the more comprehensive underlying functional association network, FunCoup.

When it comes to the choice of the underlying network, other comprehensive functional association networks exist, such as STRING (Szklarczyk et al., 2020) and HumanNet (Yang et al., 2018). The reason for using FunCoup is both because it relies solely on experimental evidence, avoiding the noise introduced by, e.g., text mining that forms the largest evidence type in STRING, and FunCoup’s superior performance when it comes to link quality compared with the other networks (Persson et al., 2021). However, networks keep evolving constantly, and their comparison is nontrivial. Therefore, the current benchmarking approach is intentionally flexible and does not require FunCoup to be used, allowing for any other network to be used in FunCoup’s place.

Performance of the studied tools was primarily assessed using ROC, AUROC, and recall. These metrics are commonly used in the field of drug repurposing. Despite being an incomplete metric by only focusing on the positive cases, the importance of recall is suggested to be favored for drug repurposing due to incomplete knowledge of all positive drug–disease combinations (Brown and Patel, 2018). The latter is also the reason why other popular metrics, such as precision, PR curves, AUPR, Matthew’s correlation coefficient, and F-measure, may be unreliably conservative for drug repurposing because they are based on the false positive rate, which may be highly overestimated. They were calculated but not included in the final tool evaluation.

Although statistically significant differences were noted between the top two methods, proximity and ANUBIX, the practical importance of one or a few percent differences is questionable. Besides, in some evaluations one tool outperformed the other while the situation was reversed in other assessments. In the end, both methods present complementary approaches contributing almost equally to a substantial proportion of unique predictions, i.e., 68.5% of all drug–disease predictions being unique to one or the other tool. This is a clear indication of the potential benefit of using the top methods in concert both for validation of predictions from one tool by the other and to get a higher coverage of the repurposable space.

Another outcome of our analysis supports the findings of the Guney2016 benchmark. The performance of drug–drug similarity–based approaches was higher than was seen for the drug–disease based ones, irrespective of the used drug repurposing method or benchmark. This could be driven by the higher recall when similarity between drug targets of approved drugs and candidate drugs is evaluated. The sets of disease genes are usually heterogeneous, containing both disease-causing genes, i.e., drivers, and genes merely affected by the disease, i.e., passengers. Drug target association with the drivers may yield a potential therapeutic effect while association with the passengers does not guarantee an effect on the disease. When association is assessed for the whole set of disease genes, the passenger genes may, therefore, introduce noise, decreasing signal-to-noise ratio and thereby also performance. A potential way to address this issue could be to purify the set of disease genes by preclustering, an approach that is shown to increase sensitivity in pathway analysis (Castresana-Aguirre, 2021). While many disease genes are known for each disease, most drugs have only one or a few drug targets, e.g., for the FDA-approved drugs in DrugBank, the median number of targets was three (Rivero-García et al., 2020). This suggests that the set of drug targets for a drug is, for the most part, a more homogeneous set. Many of the novel treatments consist of monoclonal antibodies with only one target, suggesting that even more specificity is on the horizon. This increases the signal-to-noise ratio when drug–drug similarity is used for repurposing. However, the drawback is that drug–drug similarity requires at least one drug to be approved for a disease to repurpose more drugs for that disease. On the other hand, the approach could be attempted even for diseases without approved treatments if related diseases do have drugs approved for them.

In the assessment of the potential drug repurposing candidates, it was apparent that not all the predictions were related to potential new treatment opportunities. Some drug–disease combinations were suggestive of potential drug-related side effects. This supports the fact that network-based drug repurposing methodologies can also be used to identify potential side effects, something we hope to explore in the future.

In conclusion, in this study, we employed a comprehensive framework of functional interaction data to create a platform for *in silico* assessment of network-based drug repurposing, which can be utilized for robust evaluation of new drug repurposing strategies. Additionally, we show that network crosstalk-based approaches can rival the state-of-the-art network-based repurposing method, thereby adding to the arsenal of tools that can be used by the scientific community in the search for novel applications of already approved treatments for the benefit of patients.

## Data Availability

We used R (r-project.org) version 3.6.3 for statistical tests and data visualization as well as Python (python.org). The datasets generated for this study together with corresponding code can be found in the Sonnhammer group repository https://bitbucket.org/sonnhammergroup/drugrepurposingbench/.
