# Corrigendum: Network Crosstalk as a Basis for Drug Repurposing

**DOI:** 10.3389/fgene.2022.921286

**Published:** 2022-05-16

**Authors:** Dimitri Guala, Erik L. L. Sonnhammer

**Affiliations:** ^1^ Department of Biochemistry and Biophysics, Stockholm University, Science for Life Laboratory, Solna, Sweden; ^2^ Merck AB, Solna, Sweden

**Keywords:** drug repurposing, drug repositioning, network-based, benchmark, functional association network, network crosstalk, shortest path

In the original article, there were error. The **Data Availability Statement** omitted the location of the code and its implementation.

A correction has been made to **Data Availability Statement**

We used R (r-project.org) version 3.6.3 for statistical tests and data visualization as well as Python (python.org). The datasets generated for this study together with corresponding code can be found in the Sonnhammer group repository https://bitbucket.org/sonnhammergroup/drugrepurposingbench/.

A correction has also been made to the conflict of interest statement to declare a commercial affiliation:

Author DG was employed by company Merck AB, Sweden.

The remaining authors declare that the research was conducted in the absence of any commercial or financial relationships that could be construed as a potential conflict of interest.

The authors apologize for this error and state that this does not change the scientific conclusions of the article in any way. The original article has been updated.

