# Supplementary material for: Network Crosstalk as a Basis for Drug Repurposing
Source: Front Genet. 2022 Mar 8;13:792090. doi: 10.3389/fgene.2022.792090 (PMC8958038; doi:10.3389/fgene.2022.792090)
Supplement: Supplementary file 2 [file DataSheet1.docx]

***Supplementary Material***

**
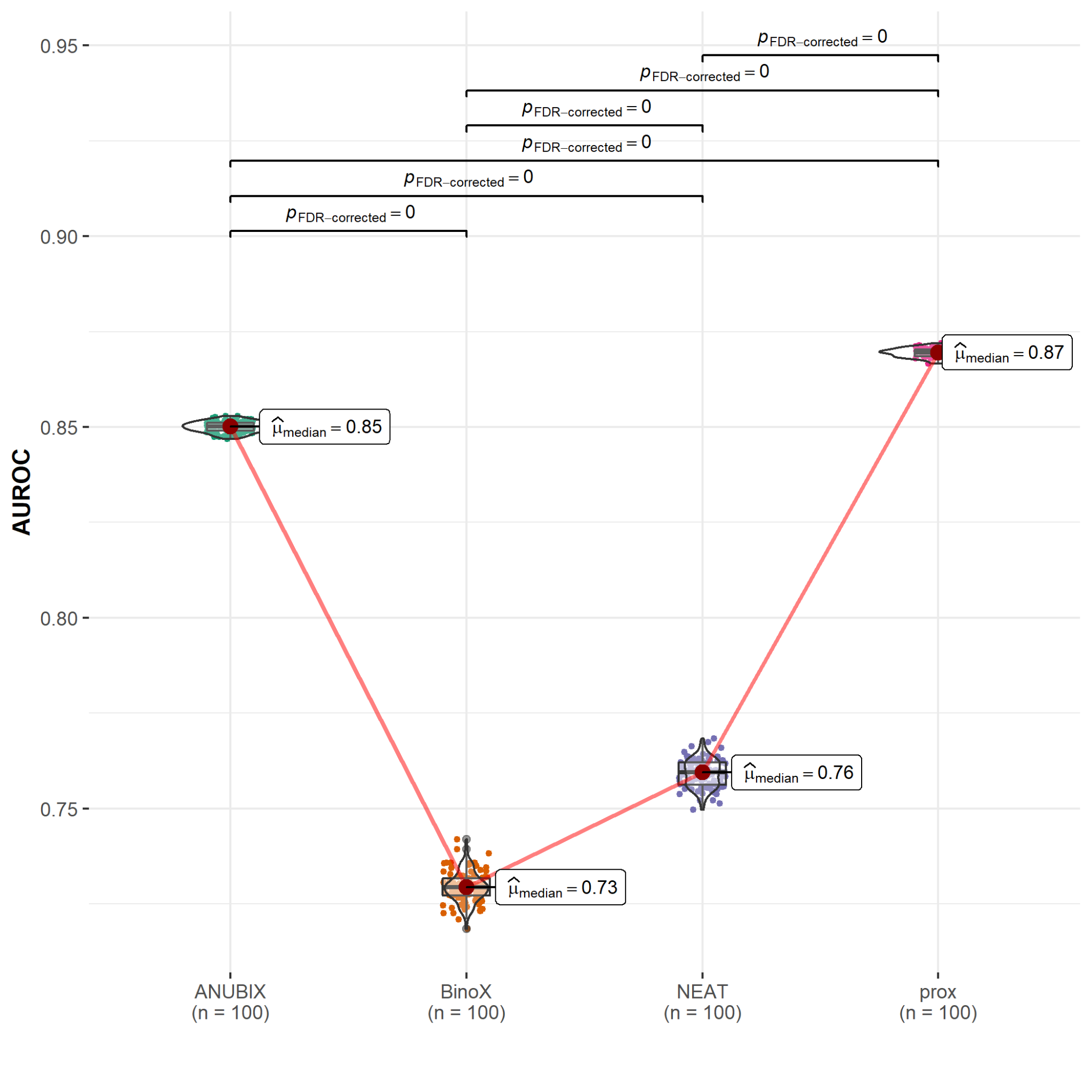
Supplementary Figure 1.** Sensitivity analysis. Drug-disease similarity-based performance on FCbench, assigning a positive label additionally to contraindicated drug-disease combinations as well as to those in clinical trials. Performance of the different drug repurposing tools: ANUBIX (green), BinoX (orange), NEAT (purple) and proximity (prox, pink) on area under ROC curve (AUROC) using sampled sets from the benchmark containing equal number of positive and negative drug-disease combinations. The pairwise Wilcoxon rank sum test was used to assess the significance of difference on AUROC. FDR-corrected p-values were obtained using the Benjamini-Hochberg procedure.


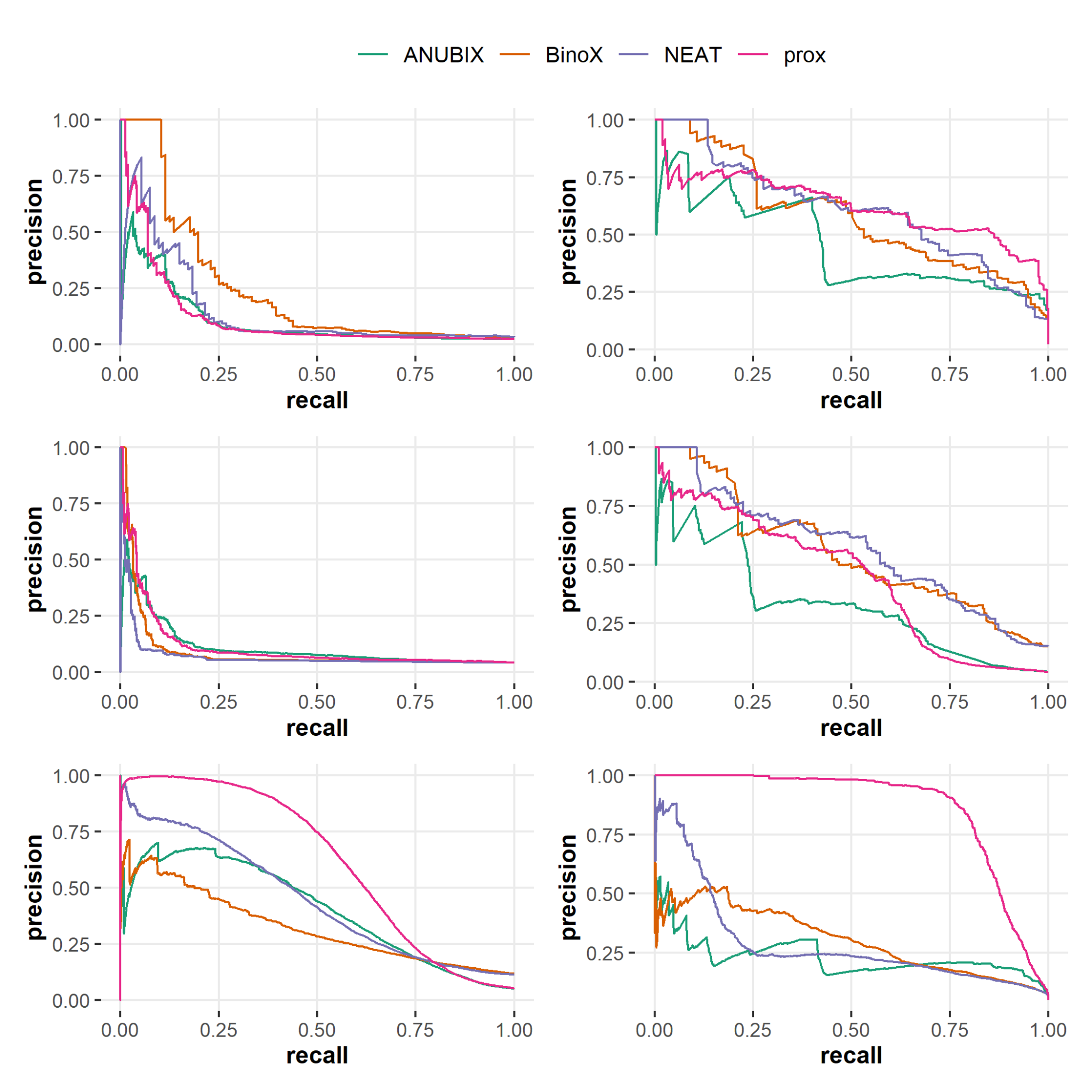
**Supplementary Figure 2.** Precision-Recall (PR) curves for the tested drug repurposing tools: ANUBIX (green), BinoX (orange), NEAT (purple) and proximity (prox, pink). PR curves for drug-disease similarity in the left column and for drug-drug similarity in the right column. A) and B) from the original benchmark, C), and D) for the time-stamped benchmark and E), and F) for FCbench.
